# Identification of Quantitative Proteomic Differences between *Mycobacterium tuberculosis* Lineages with Altered Virulence

**DOI:** 10.3389/fmicb.2016.00813

**Published:** 2016-05-31

**Authors:** Julian S. Peters, Bridget Calder, Giulia Gonnelli, Sven Degroeve, Elinambinina Rajaonarifara, Nicola Mulder, Nelson C. Soares, Lennart Martens, Jonathan M. Blackburn

**Affiliations:** ^1^Centre of Excellence for Biomedical TB Research, Witwatersrand UniversityJohannesburg, South Africa; ^2^Department of Integrative Biomedical Sciences, Institute of Infectious Diseases and Molecular Medicine, University of Cape TownCape Town, South Africa; ^3^VIB, Ghent UniversityGhent, Belgium

**Keywords:** *Mycobacterium tuberculosis*, virulence, proteomics, SRM, fitness, stress response

## Abstract

Evidence currently suggests that as a species *Mycobacterium tuberculosis* exhibits very little genomic sequence diversity. Despite limited genetic variability, members of the *M. tuberculosis* complex (MTBC) have been shown to exhibit vast discrepancies in phenotypic presentation in terms of virulence, elicited immune response and transmissibility. Here, we used qualitative and quantitative mass spectrometry tools to investigate the proteomes of seven clinically-relevant mycobacterial strains—four *M. tuberculosis* strains, *M. bovis, M. bovis* BCG, and *M. avium*—that show varying degrees of pathogenicity and virulence, in an effort to rationalize the observed phenotypic differences. Following protein preparation, liquid chromatography mass spectrometry (LC MS/MS) and data capture were carried out using an LTQ Orbitrap Velos. Data analysis was carried out using a novel bioinformatics strategy, which yielded high protein coverage and was based on high confidence peptides. Through this approach, we directly identified a total of 3788 unique *M. tuberculosis* proteins out of a theoretical proteome of 4023 proteins and identified an average of 3290 unique proteins for each of the MTBC organisms (representing 82% of the theoretical proteomes), as well as 4250 unique *M. avium* proteins (80% of the theoretical proteome). Data analysis showed that all major classes of proteins are represented in every strain, but that there are significant quantitative differences between strains. Targeted selected reaction monitoring (SRM) assays were used to quantify the observed differential expression of a subset of 23 proteins identified by comparison to gene expression data as being of particular relevance to virulence. This analysis revealed differences in relative protein abundance between strains for proteins which may promote bacterial fitness in the more virulent W. Beijing strain. These differences may contribute to this strain's capacity for surviving within the host and resisting treatment, which has contributed to its rapid spread. Through this approach, we have begun to describe the proteomic portrait of a successful mycobacterial pathogen. Data are available via ProteomeXchange with identifier PXD004165.

## Introduction

Tuberculosis disease is caused by the bacterium *Mycobacterium tuberculosis*, and remains one of the leading causes of death by a single pathogen worldwide. Despite the presence of a vaccine and a number of antibiotics for the disease, it continues to cause about 2 million deaths and 8 million new cases worldwide per year. The emergence of multiple and extremely drug resistant strains, together with HIV co-infection, are fuelling the pandemic-especially in developing countries. Furthermore, latent and sub-clinical tuberculosis infection compounds tuberculosis control strategies by creating an unseen pathogenic reservoir. Even though various strains of *M. tuberculosis* have whole genome sequences available, the bacterium still closely guards the secrets of its success as a human pathogen.

According to whole-genome analysis, members of the *M. tuberculosis* complex (MTBC) exhibit the greatest degree of genetic conservation above all other pathogenic bacteria (99.9%). This strict level of observed genetic homogeneity initially led to the assumption that genetic variety amongst different strains would not be of any clinical significance (Homolka et al., 2008). However, subsequent research has led to the understanding that traits manifested by members of the MTBC are influenced by the genetic and evolutionary background of the strains (Gagneux and Small, 2007). Although, thousands of strains have been identified, only a few seem to drive widespread disease outbreaks and multi-drug resistance (Bifani et al., 2002). *M. tuberculosis* isolates have been observed to exhibit vast discrepancies in phenotypic presentation especially with regard to clinical outcome and epidemiological behavior (Shimono et al., 2003; Baker et al., 2004; Gagneux and Small, 2007; Nicol and Wilkinson, 2008). The East Asian/Beijing *M. tuberculosis* lineage is particularly of interest due to its increasing prevalence in the global TB community, implying an apparent selective advantage compared to existing strains (Parwati et al., 2010). It is therefore cause for concern that modern Beijing lineages appear to be accumulating mutations which enhance pathogenicity, apparently under positive selection pressure (Merker et al., 2015). The exact mode by which increased pathogenicity is conferred in this lineage remains undetermined and is likely to be a combination of factors (Ribeiro et al., 2014), however some proposed mechanisms include enhanced stress response, drug resistance and altered host-pathogen interactions, as has been reviewed previously (Hanekom et al., 2011; Warner et al., 2015). On the other hand, some closely related strains in the *M. tuberculosis* complex (MTBC) have attenuated virulence in humans (such as the vaccine strain BCG), or are not typically human pathogens and will only opportunistically infect immunocompromised humans (Desforges and Horsburgh, 1991; Wang and Behr, 2014; Halstrom et al., 2015).

Whilst genetic variation across multiple strains has been studied in depth, the clinical and epidemiological consequences of genetic differences between mycobacterial strains remains poorly understood (Malik and Godfrey-Faussett, 2005). As a consequence, it is not known whether the proteome is comparatively static between different strains of *M. tuberculosis* or whether quantitative differences in the expressed proteomes could contribute in some way to differential virulence. Here, we used liquid chromatography mass spectrometry (LC-MS)-based proteomics to define and compare the proteomic complement of 6 clinically relevant mycobacterial strains within the MTBC as well as a strain of *Mycobacterium avium* as an outlier. While these strains are all pathogenic in principal, the extent to which they cause disease in humans varies greatly. We therefore aimed to identify protein expression profiles that might correlate with altered virulence amongst these strains by comparing more pathogenic strains in the MTBC to less pathogenic *Mycobacterium bovis*, BCG and *M. avium* strains.

## Materials and methods

*M. tuberculosis* isolates H37Rv, W-Beijing, CAS and LAM3 were obtained from the Medical Microbiology Division of the University of Cape Town. The clinical strains representing lineages 2 (Beijing), 3 (CAS), and 4 (LAM3/F11) were isolated from pediatric patients from Red Cross war memorial hospital, Cape Town. *M. tuberculosis* H37Rv was used in all assays as a reference strain. Phylogeny of the isolates was determined using spoligotyping and MIRU-VNTR described in Sarkar et al. (2012) and is shown in Supplementary Table 1. The Danish strain of *M. bovis* BCG was used in this study. The *M. avium* strain was obtained from the National Health Laboratory Services (NHLS) laboratory and was verified using line probe assays. *M. bovis* was obtained from Stellenbosch University Health Sciences Department in Tygerberg Hospital, Cape Town.

### Cell culture

Cells were maintained in wholly synthetic Sautons media (2% glycerol, 0.4% L-asparagine, 0.2% glucose, 0.2% citric acid, 0.05% mono-potassium phosphate, 0.05% magnesium sulfate, 0.015% Tween 80, 0.005% ferric citrate, 0.00001% zinc sulfate at pH 7.4). Briefly, 190 ml of Sautons medium was inoculated with a 10 ml starter culture (approximately 10^8^ bacteria/ml). The flasks were sealed and incubated at 37°C and 5% CO_2_ with gentle agitation until OD_600_ reached 0.9 (approximately 6 weeks).

### Protein extraction

Proteins were extracted in a Biosafety level 3 facility in line with health and safety guidelines. Briefly, the cell pellet was separated from the culture filtrate by centrifugation at 4000 × g for 15 min in a bench-top centrifuge. Cell lysis was carried out by boiling the cell pellet in 1% SDS buffer (1% SDS, 100 mM Tris-HCl pH 7.6, 0.1 mM dithiothreitol (DTT), 1 mM PMSF) for 30 min. Cell debris was separated from the protein containing supernatant by centrifugation at 10,000 × g for 15 min in a bench top centrifuge and the supernatant containing the protein was transferred into a clean tube. Protein extracts from cell lysates were concentrated and buffer exchanged to 2 M urea buffer using 3 kDa MWCO filters (Millipore). Culture filtrate proteins were concentrated and buffer exchanged into 2 M urea using 15 ml 10 kDa MWCO filters. Protein concentration was determined using the BCA assay kit (Thermo Scientific). A 10 kDa MWCO filter was used for the culture filtrate instead of a 3 kDa because this is the lowest filter size available for large volumes, however, according to manufacturer's product specifications (Millipore), proteins as low as 3 kDa are still retained on 10 kDa MWCO filters.

### Protein separation (1D SDS PAGE)

Proteins were separated according to molecular weight using an SDS PAGE gel system. The separating gels were made from 10% acrylamide: bis-acrylamide, 0.375 M Tris-HCl (pH 8.8), 7.5% SDS, 0.5% ammonium persulphate and 0.1% TEMED. The stacking gels consisted of 4% acrylamide: bis-acrylamide, 0.125 M Tris-HCl (pH 6.8), 0.1% SDS, 0.5% ammonium persulphate and 0.1% TEMED. 40 μg of each sample (culture filtrate and intracellular protein) was mixed with an equal volume of 2x sample buffer and heated at 65°C for 5 min. Electrophoresis was performed from anode to cathode at 100 V using a BioRad mini-Protean II gel system until the bromophenol blue dye reached the bottom of the gel.

### Protein visualization

Visualization of the proteins on the gel was performed using Coomasie brilliant blue R250 for 1 h (50% methanol, 10% acetic acid and 0.1% Coomasie brilliant blue R250). Destaining of the gels was carried out by incubating on a shaker overnight at room temperature in destaining solution (10% methanol, 10% acetic acid).

### In gel trypsin digestion

Each gel lane for each strain sample was divided into 5 pieces (i.e., 5 culture filtrate fractions and 5 intracellular protein fractions, hence a total of 10 fractions per strain). Each gel piece was cut into smaller cubes and washed twice with water followed by 50% (v/v) acetonitrile for 10 min. The acetonitrile was replaced with 50 mM ammonium bicarbonate and incubated for 10 min. Washes with 50 mM ammonium bicarbonate were repeated twice to remove acetonitrile. All the gel pieces were then incubated in 100% acetonitrile until they turned white, after which the gel pieces were dried *in vacuo*. Proteins were reduced with 10 mM DTT for 1 h at 57°C. This was followed by brief washing steps of ammonium bicarbonate followed by 50% acetonitrile before proteins were alkylated with 55 mM iodoacetamide for 1 h in the dark. Following alkylation, the gel slices were washed with ammonium bicarbonate for 10 min followed by 50% acetonitrile for 20 min, before being dried *in vacuo*. The proteins in the gel cubes were digested with trypsin (Promega) at 37°C overnight in a 1:50 trypsin: protein ratio. The resulting peptides were extracted twice with 70% acetonitrile in 0.1% formic acid for 30 min and then dried and stored at −20°C. Dried peptides were dissolved in 5% acetonitrile in 0.1% formic acid and 10 μl injections were made for nano-LC chromatography.

### Mass spectrometry

All experiments were performed on a Thermo Scientific EASY-nLC II coupled to an LTQ Orbitrap Velos mass spectrometer (Thermo Scientific, Bremen, Germany) equipped with a nano-electrospray source. For liquid chromatography, separation was performed on an EASY-Column (2 cm, ID 100 μm, 5 μm, C18) pre-column followed by a, EASY-column (10 cm, ID 75 μm, 3 μm, C18) column with a flow rate of 300 nl/min. The gradient used was from 5–15% B in 5 min, 15–35% B in 90 min, 35–60% B in 10 min, 60–80% B in 5 min, and kept at 80% B for 10 min. Solvent A was 100% water in 0.1% formic acid; solvent B was 100% acetonitrile in 0.1% formic acid. MS/MS data was acquired from the Orbitrap Velos in Top 20 CID mode.

### Post MS data analysis

Raw data was captured from the mass spectrometer and converted to MS2 files using MakeMS2 software (Thermo Scientific). The data was then analyzed using Crux (McIlwain et al., 2014) and Mascot (Cottrell and London, 1999), and the output of MS2PIP (Degroeve and Martens, 2013) was used additional features for the Percolator algorithm. Spectra were obtained from each fraction of the gel (a total of 5 fractions per strain) and were viewed using Peaks v5.3. The mass spectrometry proteomics data have been deposited to the ProteomeXchange Consortium via the PRIDE (Vizcaíno et al., 2016) partner repository with the dataset identifier PXD004165.

### Protein preparation for SRM-MS

Proteins were extracted in the BSL3 facility in line with health and safety guidelines as described in Section Protein extraction. Protein concentration was determined using a BCA assay according to manufacturer's protocol (#23227, Thermo Fisher Scientific). Protein preparation was performed using a filter aided sample preparation (FASP) method. Briefly, 200 μg of each protein sample was placed into a 10 KDa molecular weight cut off filter (MWCO) (Millipore). Protein cysteine residues were alkylated in the dark for 30 min in 10 mM iodoacetamide. Iodoacteamide was then removed by centrifugation at 14,000 × g for 15 min. Buffer exchange was performed twice with 8 M urea in 0.1 mM Tris-HCl pH 8.5 by centrifugation at 14,000 × g for 15 min in a refrigerated bench-top centrifuge at 18°C. The urea buffer was then exchanged for 0.05 M ammonium bicarbonate by centrifugation. Sequencing-grade modified trypsin (#608-274-4330, Promega) was added at a ratio of 1:100 enzyme: substrate and incubated overnight at 37°C in a wet chamber. The peptides were finally collected through the filter into a clean collection tube by centrifugation at 14 000 × g for 10 min.

To stop the tryptic digest the pH was lowered to 2 using 50% trifluoro acetic acid (TFA) followed by an incubation for 15 min at 37°C with shaking at 500 rpm. The peptide solution was desalted with C18 reversed-phase columns (Pierce #89870-25) according to the manufacturer's instructions. Briefly, the C18 columns were activated with 50% methanol, followed by equilibration with 5% ACN: 0.5% TFA. After loading the sample, the columns were washed 3 times with 5% ACN: 0.5% TFA. Finally, peptides were eluted with 70% ACN, dried under vacuum, and re-solubilized in 0.1% FA to a final concentration of 8 μg/μl.

### SRM mass spectrometry

All SRM experiments were performed on a TSQ Vantage triple quadrupole mass spectrometer (Thermo Fisher Scientific) equipped with a heated electrospray II ion source. For liquid chromatography and separation of peptides, a Synergi 4 μ Hydro RP 150 × 4.60 mm 80 Å pore size C18 column (serial # 630710-14) was used with a column flow rate of 300 μl/min. The gradient used was from 5–15% B in 5 min, 15–35% B in 90 min, 35–60% B in 10 min, 60–80% B in 5 min and kept at 80% B for 10 min. Solvent A was 100% water in 0.1% formic acid, and solvent B was 100% acetonitrile in 0.1% formic acid.

The mass spectrometer was operated in positive mode using electrospray ionization with a voltage of 3500 V. The capillary temperature was set to 350°C and the collision gas pressure to 1.2 mTorr. Up to 336 transitions per run were acquired with a cycle time of 3 s and a dwell time of at least 20 ms. Collision energies were calculated per individual peptide transition ion using Skyline software and further optimized by a series of energy ramping experimental steps (10 steps of 5 V) to obtain the optimum energy of each transition. MS/MS data was acquired from the CID mode. Raw data was captured from the mass spectrometer and analyzed using Skyline software.

The proteins chosen for SRM analysis were based on relevance in pathogenicity and/or virulence as stated in literature as shown in Supplementary Table 2. To design SRM assays, peptides were chosen for analysis based on their prior observation in our discovery experiments. Although, each protein was typically observed by two or more peptides in the discovery experiment, the two best performing peptides were chosen to confirm each protein in the SRM assay, with the exception of 2 proteins (Rv1818c and Rv0833) (Supplementary Table 3). Due to the small size and non-redundant nature of the *M. tuberculosis* proteome, 3 ion transitions were set as the minimum number required to identify a peptide in an SRM assay.

### SRM data analysis

Data analysis was carried out using Skyline software (MacCoss Lab software). Intra-assay ambiguity (CV) for each peptide was based on the calculated average protein concentration for a set of technical duplicate injections of each sample. Inter-assay CV was calculated for each peptide from across three biological replicates of the 7 strains. Quantitation of each peptide was carried out using the area under the curve for the peptide transitions assayed in Skyline. Retention times for the peptide standards were obtained by pre-assessment on the MS. For peptides without standards, the retention times were obtained from predictions made by Skyline software and gated at 5 s from the predicted retention time.

### Protein inference

The protein databases used to generate theoretical spectra were strain specific individual non-redundant fasta files obtained from Ensembl (www.ensembl.org), with the exception of LAM for which there is no Ensembl annotation, and W-Beijing whose annotated file is not sufficient for downstream cross strain comparison. For LAM, the UniProt fasta file was used (www.uniprot.org) and for W-Beijing, the H37Rv Ensembl fasta file was used. The parameters were standard across both searches and included carbamidomethylation of cysteine residues as a fixed modification and oxidation of methionine residues as a variable modification. Two missed cleavages were allowed and peptide mass tolerance was set at 10 ppm whilst fragment mass tolerance was set to 0.5 Da. Decoy databases were used for FDR analysis and a cut-off was set at 5% for protein identifications.

The PSMs obtained from the search were used to predict the expressed protein repertoire of each sample. MS/MS spectra from the 10 fractions per strain were searched with each individual search engine. Combining results from multiple search engines yields higher protein identifications (Shteynberg et al., 2013), and therefore all the proteins identified from each individual search engine were combined and redundancy was removed to give one complete non-redundant dataset per study organism.

## Results

Total protein extracts from the discovery MS approach were quantified using BCA assay with concentrations ranging between 2.5–15 μg/μl for total cellular proteins and 10–40 μg/μl for culture filtrate proteins. Each tryptically digested sample was analyzed on the Orbitrap Velos to produce an LC-MS and MS/MS dataset. To assess the efficiency of the tryptic digest a descriptive analysis software package in Protein Pilot was used and the results are summarized in Supplementary Table 4.

As shown in Supplementary Figure 1A, the MS1 scan of H37Rv confirms a successful tryptic digest with the total ion chromatogram in showing a steady elution of peptides across the LC gradient. The 2D MS chromatogram (Supplementary Figure 1B) demonstrates the complexity of the sample, showing a significant number of discrete tryptic peptides eluting at the marked time point indicated on the 1D chromatogram (~2600 s).

For the analysis of the MS2 spectra a software pipeline was implemented that combines the results of different peptide identification strategies. At the core of our pipeline is the semi-supervised learning algorithm implemented in Percolator (Brosch et al., 2009) that has been shown to obtain high identification sensitivity. The first tool in the pipeline is Crux (McIlwain et al., 2014) which is a reimplementation of the popular tool Sequest with added post-processing by the Percolator algorithm. The second tool is Mascot (Cottrell and London, 1999) for which again Percolator was used to post-process the MOWSE identification scores^1^.

The Percolator tool allows for adding new features that can be exploited by the semi-supervised learning algorithm to further increase peptide identification sensitivity. It has been shown that adding features obtained from MS2 peak intensity predictions can significantly increase sensitivity (Sun et al., 2007). Therefore, we employed the MS2PIP (Degroeve and Martens, 2013) tool to predict the b- and y-ion peak intensities for all peptides suggested by Mascot (top ranked peptide for each MS2 spectrum). We then computed several features from the difference between the predicted and the observed MS2 peak intensities, such as the Pearson correlation. We observed that adding these features to the Percolator algorithm for Mascot did indeed increase identification sensitivity significantly.

**Figure 1:**
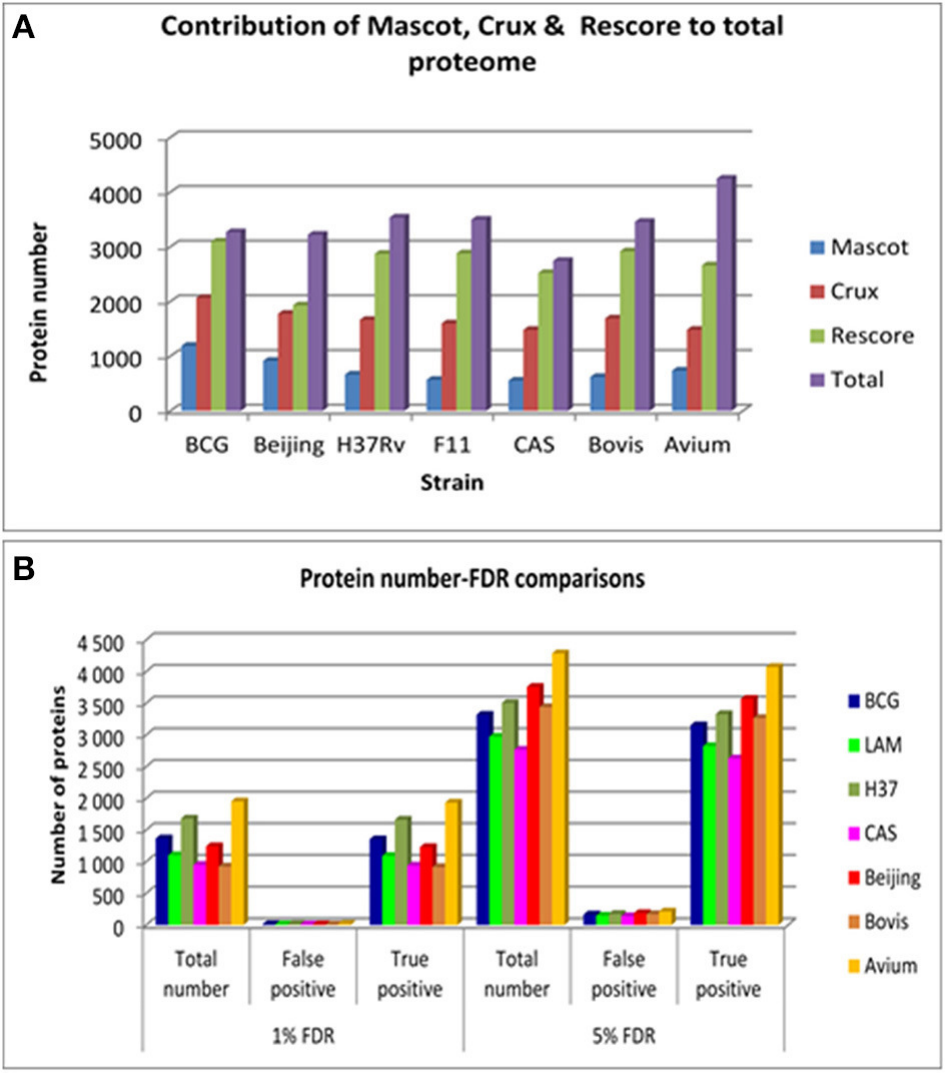
**(A)** The contribution of each of the search engines to the total number of non-redundant proteins obtained per strain at 5% FDR. Mascot is shown in blue bars, Crux is shown in red bars, Mascot+MS2PIP+Percolator is shown in green bars and the total non-redundant library is shown in purple bars. **(B)** The comparison between 1 and 5% FDR across all strains. This illustrates the total number of proteins obtained for each strain at each FDR, and the proportion of true and false positives in that FDR bracket.

Comparison of protein numbers obtained at 1% and 5% FDR showed that the use of 5% FDR allows a substantial increase in the absolute number of true positives with an insignificant increase in the absolute number of false positives, hence providing an apparently favorable trade-off in true positives over false positives (Figure 1). The final proteome obtained from each strain using all algorithms represented a high proportion of the theoretical protein fasta files for each strain, as shown in Table 1.

**Table 1:** **Total non-redundant number of proteins obtained in the experiment compared to the total number of proteins in the theoretical fasta file**.

**Strain**	**Total # proteins in fasta file**	**Total # detected experimentally (5% FDR)**	**% of total theoretical proteome found**
*M. avium*	5314	4250	80
*M. tuberculosis H37Rv*	4049	3539	87
*M. tuberculosis Beijing*	4049	3224	80
*M. tuberculosis CAS*	4049	2746	68
*M. tuberculosis LAM*	3904	3500	90
*M. bovis BCG*	4041	3272	81
*M. bovis*	4001	3461	87

### Data alignment for downstream comparison

**Figure 2:**
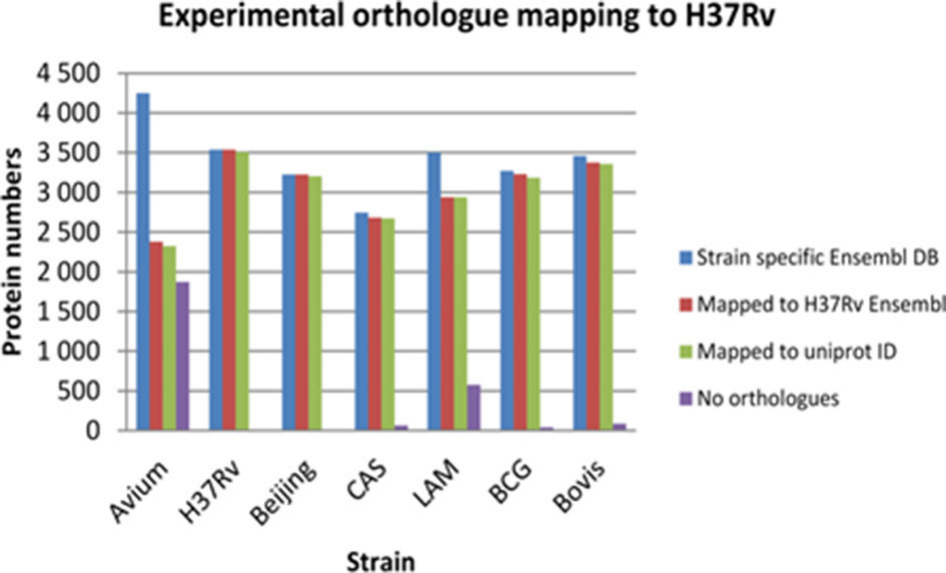
**The losses in number of identified proteins obtained after experimental ortholog mapping to ***M. tuberculosis*** H37Rv for each strain**. *Avium*—*Mycobacterium avium*, H37Rv—*Mycobacterium tuberculosis* H37Rv, Beijing—*Mycobacterium tuberculosis* W. Beijing, CAS—*Mycobacterium tuberculosis* CAS, LAM—*Mycobacterium tuberculosis* LAM, BCG—*Mycobacterium bovis* BCG, Bovis—*Mycobacterium bovis*.

To carry out an effective cross strain comparison, it was crucial to ensure that as much of the theoretical proteome as possible was observed by discovery MS. After obtaining a non-redundant dataset for each strain using strain specific databases, it became necessary to convert all the protein IDs into a standard protein ID by orthology mapping to allow effective cross strain comparison. To achieve this, the total non-redundant IDs from each strain obtained by searching against its individual Ensembl fasta file were then mapped back to the Ensembl H37Rv protein IDs. These were all in turn mapped to UniProt accession numbers and Tuberculist “Rv” loci numbers to facilitate downstream analysis with tools such as GO analysis and pathway mapping. It was observed that there is a slight deficiency in ortholog mapping data between databases (Ensembl and UniProt), as well as shortcomings in ortholog mapping between strains. These discrepancies lead to a minor loss of information, as represented in Figure 2. The discrepancy in ortholog mapping was much more pronounced when mapping protein IDs to H37Rv from the more distantly related *M. avium*, which lies outside the MTBC group; this resulted in loss of approximately 50% of the biological information in downstream comparisons to *M. avium*. Other strains showed relatively small losses in the number of experimentally observed proteins mapped to H37Rv orthologs, for instance the LAM strain had approximately 600 proteins with no orthologs in H37Rv which were therefore not included in the cross-species comparison.

### Qualitative cross species comparison

**Figure 3:**
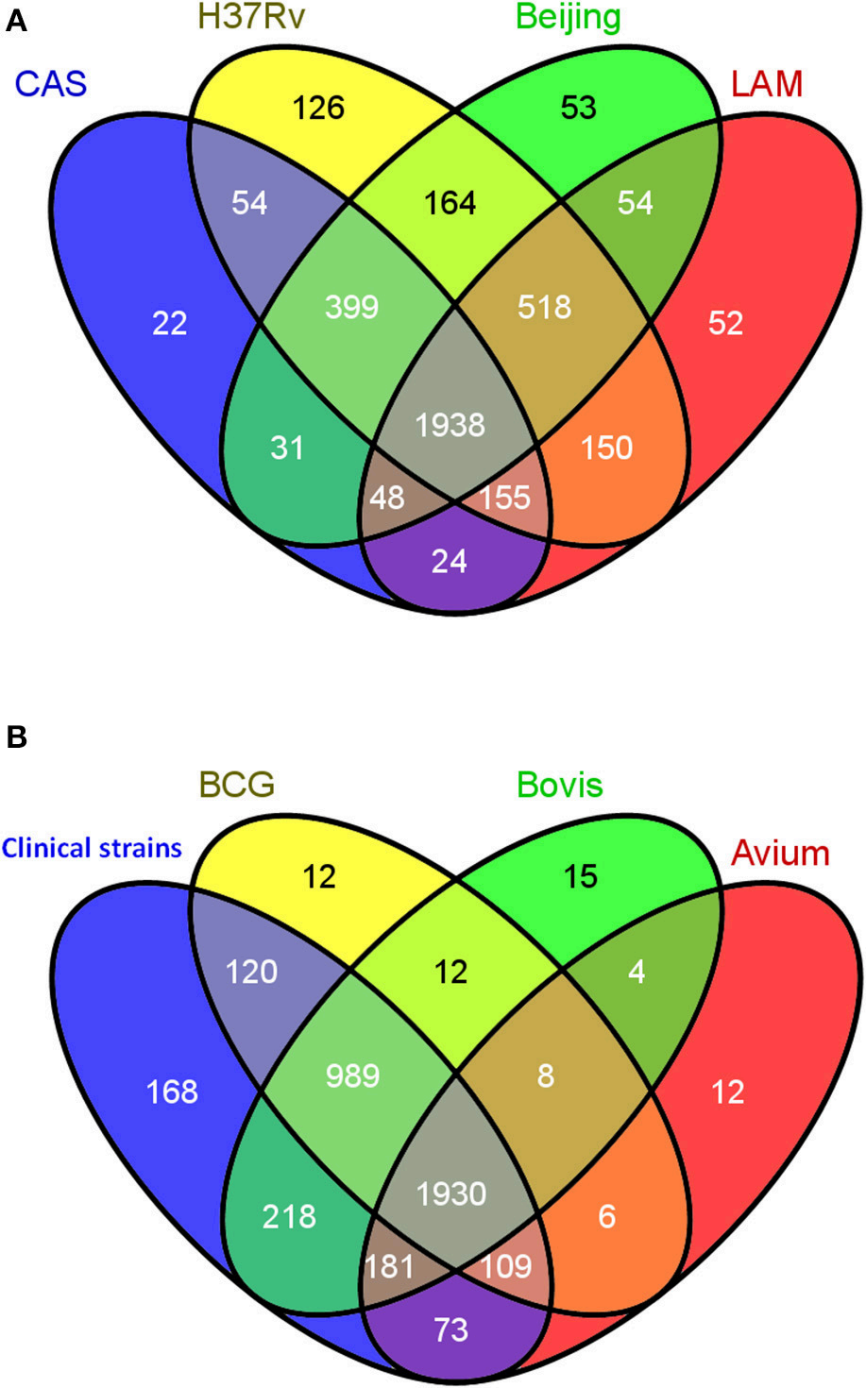
**Qualitative cross strain/species data analysis. (A)** Venn diagram showing the overlap in the numbers of protein identifications between the 4 strictly *Mycobacterium tuberculosis* strains CAS, LAM, W-Beijing, and H37Rv. **(B)** Venn diagram representing the comparison between the collective non-redundant proteins obtained in the 4 strictly *M. tuberculosis* strains (CAS, LAM, W-Beijing, and H37Rv) denoted “Human clinical strains” and those of *M. bovis*, BCG, and *M. avium*.

With congruent IDs, strains were cross-compared to obtain a comprehensive qualitative comparison as summarized in Figure 3 using the Venn diagram tool Venny [267]. Protein IDs from the 4 *Mycobacterium tuberculosis* strains were compared (Figure 3A) and, as expected, the majority of observed proteins were found to be shared amongst all *M. tuberculosis* strains, with less than 5% being strain specific. A total of 1938 proteins comprise the shared proteins among the 4 *M. tuberculosis* strains, perhaps representing a *M. tuberculosis* core proteome. A second diagram was generated comparing the collective, non-redundant proteomes of the four *M. tuberculosis* strains to those of *M. bovis*, BCG, *M. avium* (Figure 3B). Surprisingly, *M. avium* had many proteins in common with the MTBC strains, with only 12 unique proteins apparently unique to *M. avium*; this may simply reflect though the deficiencies in ortholog mapping between more distantly related organisms. The four *M. tuberculosis* strains also share 989 common proteins with *M. bovis* and BCG which they do not share with *M. avium*. A group of 168 was observed uniquely in the *M. tuberculosis* strains and not found in *M. bovis*, BCG or *M. avium*; these proteins were therefore earmarked as candidate virulence factors to be further explored.

### Protein expression profiling

We subsequently sought to quantitatively assess a subset of the candidate virulence factors identified by discovery MS and cross-strain comparison using selected reaction monitoring (SRM)—a sensitive, reproducible and quantitative MS technique. Since the design of SRM robust assays can be a lengthy process and the capacity to highly multiplex hundreds of SRM assays remains challenging, we devised a strategy to create a short list of candidate proteins with possible relevance in differential clinical phenotypes observed between the *M. tuberculosis* isolates for subsequent quantitative proteomic analysis. To do this, we compared our proteomic data on each of the 168 proteins (Supplementary Table 5) that were observed only in the *M. tuberculosis* strains with 771 gene expression data sets contained in the TBDB, representing varying *in vitro* models of TB disease. We focussed attention in particular on 7 categories of experiment from the TBDB that aimed to more closely reflect *in vivo* conditions (e.g., starvation models, macrophage infection models, hypoxic models, etc.), the logic being that consistent over-expression of a protein in one of those categories might plausibly confer a selective advantage to the bacterium *in vivo*; the categories chosen are listed in Table 2.

**Table 2:** **The number of significantly differentially expressed proteins from the 168 proteins unique to human clinical strains segregated into over- or under-expressed per category assessed (www.tbdb.org)**.

**TBDB Experiment**	**Over-expressed**	**Under-expressed**
Acid media	58	38
Macrophage	18	53
Hypoxia	59	49
NO treatment	78	32
Starvation	51	26
Persistence	23	43
Antibiotics	40	54

**Figure 4:**
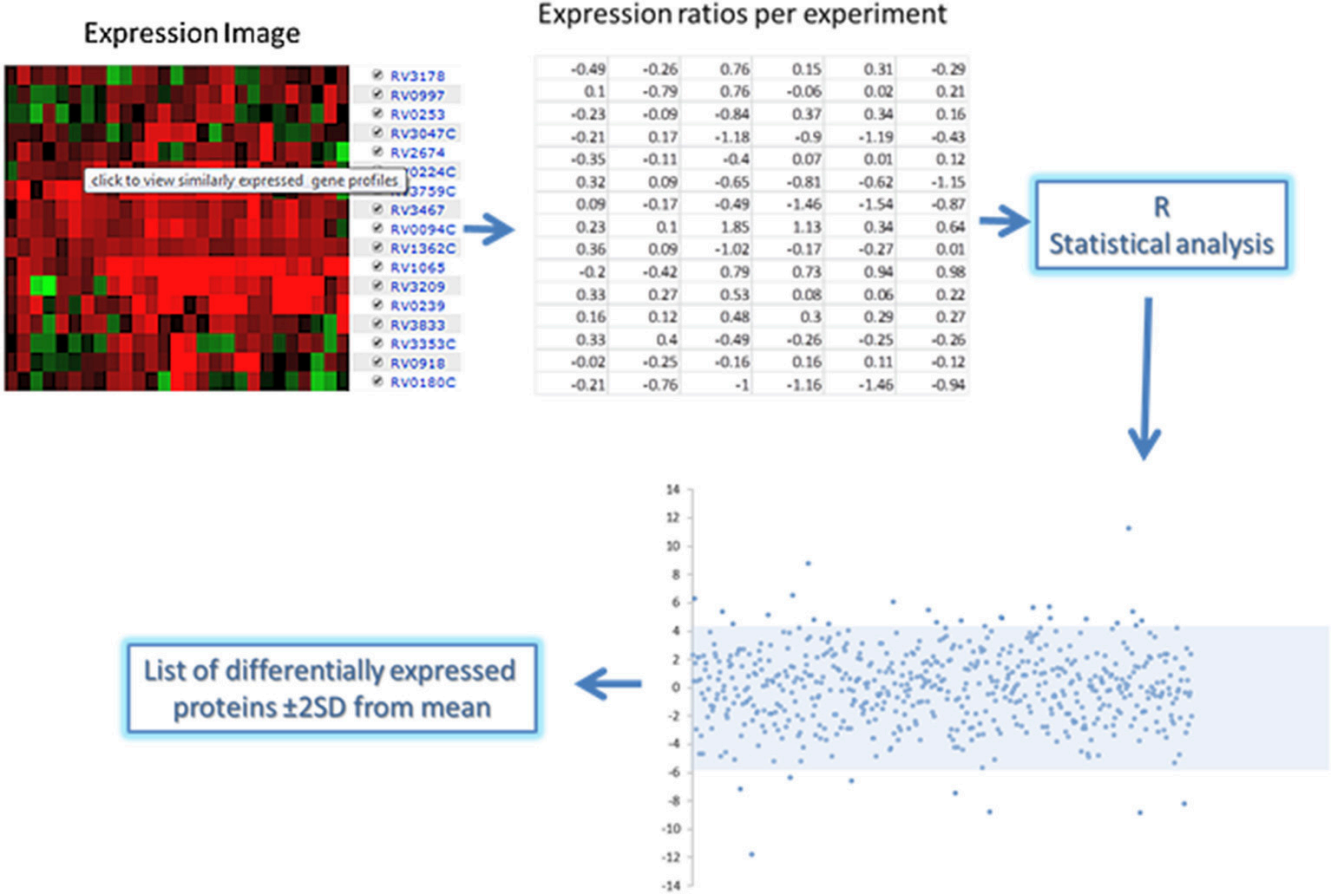
**Gene expression analysis approach of the short list of 168 proteins expressed in human clinical strains**. Expression ratio data was obtained from the chosen TBDB experiments and statistical packages in R were used to convert the ratios into fold changes. Analysis of the fold changes was done in R to indicate proteins that have significant fold changes (≥2 from the mean) which are shown in the scatterplot above and below the shaded area.

For each of the 168 proteins, we assessed whether they were significantly over- or under-expressed in each of the 7 chosen categories of gene expression models and carried out statistical analysis on the gene expression values obtained from the TBDB experiments using packages in R (strategy depicted in Figure 4). Proteins from the 168 protein set whose gene expression showed an average fold change of ≥2 SD from the mean across all datasets in an individual condition were taken as significantly differentially expressed in that condition. Proteins that had significant fold change for fewer than 4 out of the 7 categories were removed from this list, resulting in a final shortlist of 23 proteins, summarized in Table 3. In order to create SRM assays for the shortlisted proteins, the *M. tuberculosis* proteome library (Schubert et al., 2013) was consulted and validated SRM assays for the proteins of interest were extracted.

**Table 3:** **The 23 mycobacterial proteins identified in discovery proteomic experiments which were subsequently selected for quantitative targeted proteomic analysis**.

**Rv locus**	**Function and prediction from gene expression data**	***p*-value**
Rv0301	VapC2; toxin; possible mRNAse; impaired growth when expressed | macrophage infection models	6,75E-07
Rv0899	ArfA; outer membrane porin A; tolerance to acidic conditions, impaired growth at pH 5.5	5,55E-08
Rv0901	ArfC; unknown function; tolerance to acidic conditions, impaired growth at pH 5.5	6,42E-06
Rv0966	Unknown function; highly activated in the early stages of tuberculosis blood brain barrier invasion (CNS TB)	1,96E-05
Rv1002c	Pmt; probable mannosyltransferase; conserved membrane protein growth and survival in host	9,35E-07
Rv1346	MbtN; mycobactin biosynthesis; adaptation to intracellular environment | stress response models	0,020716
Rv1380	PyrB; pyrimidine biosynthesis; essential for growth; high confidence drug target | drug response models	0,039441
Rv1381	PyrC; pyrimidine biosynthesis; growth and survival in host | drug response models	4,59E-09
Rv1383	CarA; pyrimidine biosynthesis; growth and survival in host | drug response models	1,08E-06
Rv1384	CarB; pyrimidine biosynthesis; growth and survival in host	4,47E-09
Rv1980c	Mpt64; Unknown function; tolerance to starvation, highly immunogenic; vaccine and drug target potential	0,422015
Rv1997	CtpF; Metal cation-transporting ATPase; implicated in dormancy/persistance, response to hypoxia, NO	6,10E-06
Rv2108	PPE36; unknown function; immuno-active membrane component; diagnostic and vaccine target	0,876293
Rv2126c	PE_PGRS37; unknown function; possible virulence/adaptation	0,988059
Rv2136c	UppP; undecaprenyl pyrophosphatase; high confidence drug target | host immune evasion and virulence models	5,32E-12
Rv2156	MraY; peptidoglycan biosynthesis; growth and survival in host, high confidence drug target	0,97868
Rv2703	SigA; primary sigma factor in *M. tuberculosis*; host immune response modulator, virulence, growth in host, high confidence drug target | host immune evasion and virulence models	0,026023
Rv3340	MetC; methionine biosynthesis; growth and survival in host	0,649041
Rv3412	Unknown function; hypothetical protein; essential for cholesterol metabolism, essential during infection	3,27E-07
Rv3621c	PPE65; unknown function; possible virulence/adaptation	0,035777
Rv3709c	Ask; asparate kinase; survival in host	1,23E-05
Rv1818	PE-PGRS33; unknown function; modulation of host immune response, response to oxygen and starvation	1,61E-06
Rv0833	PE_PGRS13; unknown function; possible virulence/adaptation	9,79E-08

A minimum of 2 peptides per protein and 3 transitions per peptide were assessed by SRM for the shortlisted proteins in all seven strains. Intra-assay and inter-assay coefficients of variation were determined for each individual peptide. Intra assay variability was based on 3 technical replicates per strain whilst inter assay variability was assessed based on 2 biological replicates. The signal of each peptide observed was obtained by summing the peak areas of each measured transition for that peptide and then normalizing by the total number of cells per strain at the point of protein extraction. An ANOVA test was used to determine if there was a significant difference in the expression of each of the peptides representing each protein. The single factor ANOVA results (Table 3) shows that of the 23 peptides assessed, 18 had a significant difference in expression between the 7 strains. The proteins were then broadly classified into 4 groups denoting some aspect of the organisms' success in the host (Supplementary Figure 2) in order to aid further interpretation of the data.

The four proteins assayed with roles in drug response are PyrB, PyrC, CarA, and CarB, and all form part of the pyrimidine biosynthetic operon in *M. tuberculosis*. All with the exception of carB (Rv1384) are more abundant in *M. avium* compared to the other strains, but within the MTBC the Beijing strain has the highest expression of PyrB, PyrC, and CarA.

Amongst proteins which are known to modulate the host immune response, the uncharacterized hypothetical protein Rv0966 is highly expressed in the LAM strain compared to the other strains. In this functional category, Rv2136c, Rv1002, Rv2703, and Rv2108 are more abundant in the Beijing strain. Rv1818c appears to be more abundant in the CAS strain while in the other strains it is present at comparatively low amounts.

Amongst proteins responsible for the growth of *M. tuberculosis* in the host, a possible toxin with unknown function, VapC2 (Rv0301), has the highest relative expression in the Beijing strain. PE_PGRS13 (Rv0833), MetC (Rv3340), and conserved hypothetical protein Rv3412 are all more abundant in the LAM strain than in BCG or *M. avium*, although similarly highly abundant in H37Rv and *M. bovis*. PPE65 (Rv3621c) appears to be upregulated in both LAM and *M. avium* strains.

In terms of adaptation to stress, the Acyl-CoA dehydrogenase MbtN (Rv1346) is particularly abundant in the Beijing strain. The protein Rv0901 is apparently less abundant in *M. bovis* and BCG strains whereas it is relatively abundant in the other strains, particularly so in H37Rv and *M. avium*. Aspartate kinase (Rv3709c) is more abundant in H37Rv and LAM strains comparatively, and almost entirely absent in *M. avium* and *M. bovis*.

**Figure 5:**
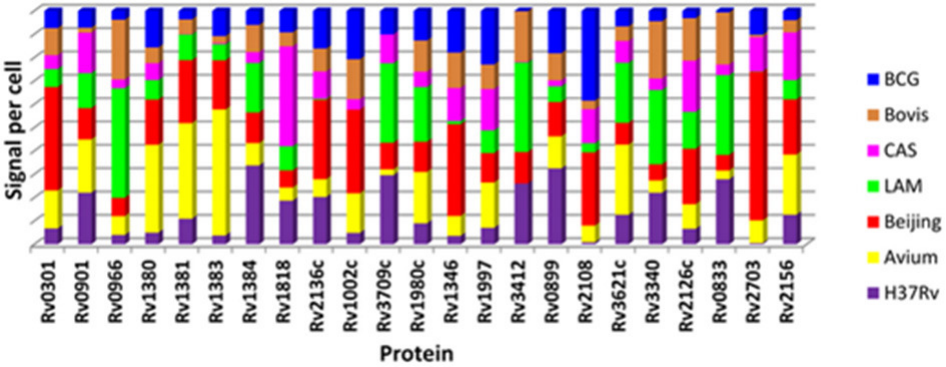
**Comparison of relative protein quantity per strain for the 23 mycobacterial proteins as determined by SRM**. Each color in the stacked bar represents the percentage contribution of that strain to the total detected amount of each protein.

The contribution to total signal detected for each protein as measured by SRM in each of the 7 strains is represented in Figure 5. This analysis demonstrates that proteins which were detected in a particular strain in the discovery experiment, e.g., Rv0301 and Rv1002c which were observed only in the Beijing strain, tend to contribute the highest signal when measured by SRM. This appears to be clearly the case for 14 of the 23 proteins assessed (Rv0301, Rv1381, Rv1383, Rv1002c, Rv3709c, Rv1346, Rv3412, Rv2108, Rv3621, Rv0833, Rv0966c, Rv1818c, Rv2136c, and Rv3340).

## Discussion

Although, genetically similar, different strains of *M. tuberculosis* present very different clinical phenotypes in terms of virulence. We therefore postulated that there may be a proteomic mechanism underpinning the differences in pathogenicity observed between strains of *M. tuberculosis* and we explored this possibility using discovery and targeted mass spectrometry techniques. In order to reveal candidate proteins that might be involved in differential pathogenicity, we aimed to compare proteomes between individual pathogenic and non-pathogenic mycobacteria, noting that both BCG and *M. avium* can cause TB-like disease in immune compromised individuals, suggesting that their pathogenicity is attenuated, not lost entirely. To underpin our intended cross-strain and cross-species proteomic comparisons, we first carried out an exhaustive mass spectrometry-based discovery proteomics analysis of the 7 mycobacterial strains. Through use of a sophisticated bioinformatic strategy, combining data from multiple search engines, we obtained >80% coverage at the protein level for 6 out of the 7 theoretical proteomes. Combining data across the 4 *M. tuberculosis* strains, we identified a total of 3788 *M. tuberculosis* proteins with high confidence, meaning that we failed to observe only 235 out of the predicted 4023 proteins in the *M. tuberculosis* proteome. To our knowledge, this level of discovery proteomic coverage across multiple *M. tuberculosis* strains is unprecedented, although we note that recently reported SWATH-based analyses on *M. tuberculosis* H37Rv have come close to this figure (Schubert et al., 2015). Surprisingly, although the 23 proteins subsequently quantified by SRM were initially observed only in the *M. tuberculosis* strains by discovery MS analysis, they were in fact all identified in all 7 strains by SRM analysis, albeit with relative quantifications that correlated to a large degree with our discovery data. Furthermore, SRM analysis of 45 predicted *M. tuberculosis* proteins that had not been observed in our discovery MS analysis of any of the 7 mycobacterial strains revealed that half were in fact expressed in each of the *M. tuberculosis* strains (data not shown), presumably reflecting low absolute expression levels for those proteins, below the detection limit for discovery MS. Taken together, our data suggests that the total expressed complement of proteins is remarkably similar in the different clinical strains of *M. tuberculosis* and moreover that virtually the entire *M. tuberculosis* proteome is expressed in all strains, at least under optimal *in vitro* conditions. However, our data also clearly demonstrates that significant, quantitative differences in expression levels exist between strains which may directly influence the phenotype of these strains. While it is possible that protein quantity does not track with the enzyme activity of that protein in a cell, due to allosteric effects or post-translational modifications, enzyme activity was not measured in our study.

Cross-strain and cross-species comparisons of our semi-quantitative discovery proteomics data were limited only by the relatively poor ortholog mapping found between *M. avium* and *M. tuberculosis* H37Rv and enabled identification of 168 proteins that were originally observed only in the *M. tuberculosis* strains and were therefore considered candidates that might contribute to the differential virulence of these mycobacterial strains. However, we were conscious that our proteomic data had been generated under one set of culture conditions that were likely far removed from the true host environment in TB disease. We therefore cross-correlated our proteomic data with 771 gene expression models deposited in the TBDB, covering a wide range of different *in vitro* culture conditions and exogenous stresses on *M. tuberculosis* that can be thought of as mimicking various aspects of the host environment (e.g., macrophage infection; hypoxia; starvation; etc.). The genes for 23 of the 168 proteins were found to be significantly up- or down-regulated in macrophage-based and related *in vitro* gene expression models of TB disease and we therefore carried out quantitative analysis of their protein expression *in vitro* across the 7 strains, focussing particularly on differential expression in the Beijing and LAM lineages that are known to have particularly virulent clinical phenotypes (Pillay and Sturm, 2007; Cowley et al., 2008).

One of *M. tuberculosis*'s many features as a pathogen is its ability to evade the host innate and acquired immune responses such that it is capable of attaining latency and can potentially remain relatively quiescent in alveolar macrophages for decades (Flynn and Chan, 2003). Here, we identified four proteins which modulate the host immune response which are significantly more abundant in the Beijing strain compared to other strains and may therefore have functional significance in conferring virulence in *M. tuberculosis—*Rv2136c, Rv1002c, Rv2703, and Rv2108. Mutant *M. tuberculosis* with insertionally inactivated Rv2136c, a known virulence factor of the MTBC (Forrellad et al., 2013), has severe hypersensitivity to acid and a number of other stresses (Vandal et al., 2009). Rv2136c (uppP) is an undecaprenyl pyrophosphate phosphatase which recycles undecaprenyl pyrophosphate back to undecaprenyl phosphate so that it can act again as a receptor for the UDP-MurNAc-pentapeptide to make C55-PP-MurNAc-pentapeptide (lipid 1). The antibiotic Bacitracin inhibits peptidoglycan synthesis by sequestering undecaprenyl diphosphate, thereby reducing the pool of lipid carrier available, whilst increased expression of uppP provides resistance; by extension, Rv2703c might therefore be involved in virulence by speeding up the recycling of key lipid intermediates and hence cell wall biosynthesis, thus conferring a selectable advantage on the virulent Beijing strains. Similarly, sigma factor A (sigA), Rv2703, is the primary sigma factor in this bacterium and is essential for growth. Increased initiation of transcription, and thus RNA processing capacity, may therefore be another mechanism by which this strain has achieved hypervirulence, perhaps coupled to the observed increased expression of several proteins involved in pyrimidine biosynthesis (pyrB, pyr C, CarA). Although the function of Rv1002c is unknown, it is essential for growth in H37Rv (Sassetti et al., 2003), whereas the PPE family protein PPE36 (Rv2108) has no known function and is non-essential for growth in H37Rv. Both of these proteins therefore represent attractive targets for further investigation.

Once inside the host macrophage, *M. tuberculosis* becomes dependent on the intracellular environment for sources of carbon (McKinney et al., 2000; Eisenreich et al., 2010) and iron. The acyl-CoA dehydrogenase, MbtN (Rv1346), is involved in the production of mycobactins which are thought to be vital for the acquisition of iron within the macrophage and are therefore considered to be virulence factors (De Voss et al., 2000). It is notable therefore that MbtN protein was significantly more abundant in the Beijing strain, suggesting that this strain's capacity to acquire iron intracellularly may be superior. The capacity to produce essential amino acids in the host may also provide a selective advantage in terms of virulence, as demonstrated by the increased relative abundance of the O-acetylhomoserine sulfhydrylase MetC (Rv3340) and aspartate kinase Ask (Rv3709c) in the LAM strain.

Another important feature of intracellular *M. tuberculosis* in the persistent phase is the toxin/antitoxin system, of which *M. tuberculosis* has a remarkable 79 encoded loci (Sala et al., 2014). Here we found that the possible toxin VapC2 (Rv0301) was significantly more abundant in the Beijing strain than other clinical strains. Vap C has been reported to suppress translation by hydrolysis of mRNA, so its increased expression in the Beijing strain may represent an evolutionary advantage by providing an efficient means to erase previous transcriptional profiles, thus allowing *M. tuberculosis* to rapidly reprogram the proteome and hence change the metabolic state of the cell in response to rapidly changing external stresses during the bacterium's host-based lifecycle.

Finally, proteins with unknown function are clear targets for further investigation—for example the PE_PGRS and PPE family proteins which are known virulence factors yet currently have no functional categorization. Both PE_PGRS13 (Rv0833) and PPE65 (Rv3621c) as well as conserved hypothetical proteins Rv0966 and Rv3412 are abundant in the LAM strain. Interestingly, Rv0901—a possible exported or membrane protein with unknown function—is abundant in all measured strains except for *M. bovis* and BCG, both of which have attenuated pathogenicity. The loss of this protein could therefore alter the pathogenicity of the bacterium, indicating that this protein is a potential therapeutic target.

An important caveat for any quantitative *in vitro* study on *M. tuberculosis* that aims to correlate gene or protein expression levels with *in vivo* clinical phenotypes is that significant differences in expression observed under specific *in vitro* conditions may not accurately reflect the situation at the site of disease due to altered environmental influences on expression. This is further compounded by the fact that it is not currently technically possible to isolate sufficient *M. tuberculosis* bacilli from the site of disease in a human lung for a discovery proteomics experiment and by the fact that the clinical definitions of “virulence” and “pathogenicity” are themselves largely qualitative. In order to mitigate this caveat, we therefore correlated our quantiative proteomic data with over 700 gene expression models of TB disease—themselves acquired under a number of different *in vitro* conditions that each mimic in some way aspects of the stress likely to be experienced by *M. tuberculosis* at the site of disease—in order to provide a logical means to infer biological significance from *in vitro* data with greater confidence. A testable prediction from the *in vitro* quantitative proteomic data presented here is thus that the observed differential expression of specific mycobacterial proteins across these 7 strains when cultured under a common set of environmental conditions will affect clinical phenotype *in vivo*. However, the true role of these proteins in virulence will need to be validated in due course by targeted analysis of limiting numbers of *M. tuberculosis* bacilli isolated from the site of disease.

## Conclusion

Through our combined discovery- and quantitative proteomic analysis of differential protein expression in 7 mycobacterial strains of varying pathogenicity and virulence, we have uncovered previously unknown, statistically significant quantitative differences in the expression of numerous proteins which begin to shed new light on differential virulence in *M. tuberculosis* strains. In particular, our data suggests strain specific bacterial fitness in the W-Beijing lineage, including: the ability to rapidly remodel the *M. tuberculosis* proteome in response to altered environments; up-regulation of key sigma factors to support rapid transcriptional responses; up-regulation of enzymes involved in pyrimidine biosynthesis and cell wall biosynthesis to promote rapid growth; enhanced mycobactin biosynthesis to promote iron scavenging in the host. These individually selectable traits may then conceivably work together to provide the W-Beijing lineage with an enhanced ability to establish primary infection and active TB disease in a new host. These are testable hypotheses and further research is underway on this now.

## Author contributions

JP carried out research, wrote initial draft. BC analyzed data, refined and rewrote initial draft to produce final draft. GG, SD, ER, NM, NS, LM assisted with data analysis and JB is principal investigator.

### Conflict of interest statement

The authors declare that the research was conducted in the absence of any commercial or financial relationships that could be construed as a potential conflict of interest.

## Footnotes

^1^http://www.sanger.ac.uk/science/tools/mascotpercolator
