# Supplementary material for: Identification of Quantitative Proteomic Differences between Mycobacterium tuberculosis Lineages with Altered Virulence
Source: Front Microbiol. 2016 May 31;7:813. doi: 10.3389/fmicb.2016.00813 (PMC4885829; doi:10.3389/fmicb.2016.00813)
Supplement: Supplementary file 1 [file Table1.DOCX]

Supplementary Table 1: Genotype of selected *Mycobacterium tuberculosis* strains. Spoligotyping octal codes and MIRU-VNTR numbers for the strains under investigation in the study adapted from Sarkar et al (2012) (16).

|  | **Spoligotype** | **MIRU-VNTR** | **Lineage** |
| --- | --- | --- | --- |
| **H37Rv** | 777777477760771 | 243132253233552 | Lineage 4 (Laboratory strain) |
| **W-Beijing** | 000000000003771 | 442335464485372 | Lineage 2 (clinical strain) |
| **CAS 1** | 702777740003771 | 4423664??285373 | Lineage 3 (clinical strain) |
| **LAM3/F11** | 774377007760771 | 442365542253173 | Lineage 4 (clinical strain) |
