# Supplementary material for: Identification of Quantitative Proteomic Differences between Mycobacterium tuberculosis Lineages with Altered Virulence
Source: Front Microbiol. 2016 May 31;7:813. doi: 10.3389/fmicb.2016.00813 (PMC4885829; doi:10.3389/fmicb.2016.00813)
Supplement: Supplementary file 4 [file Table4.DOCX]

Supplementary Table 4: Protein pilot descriptive statistics summary showing MS/MS breakdown of the tryptic digest of H37Rv. The table shows that the majority of spectra (80.4%) contained no missed cleavages. Overall, 99.6 % of the spectra had a maximum of two missed cleavages whilst 0.2 % had more than 3 missed cleavages, a trend which was similar in tryptic digests of all other strains.

| Missed Cleavages | Spectra in selected set | % of selected set |
| --- | --- | --- |
| 0 | 3695 | 80.4% |
| 1 | 865 | 17.5% |
| 2 | 84 | 1.7% |
| 3 | 6 | 0.1% |
| 4 | 7 | 0.1% |
| 5 | 2 | 0.01% |
