# Supplementary material for: Identification of Quantitative Proteomic Differences between Mycobacterium tuberculosis Lineages with Altered Virulence
Source: Front Microbiol. 2016 May 31;7:813. doi: 10.3389/fmicb.2016.00813 (PMC4885829; doi:10.3389/fmicb.2016.00813)
Supplement: Supplementary file 6 [file Image1.PDF]

(a)

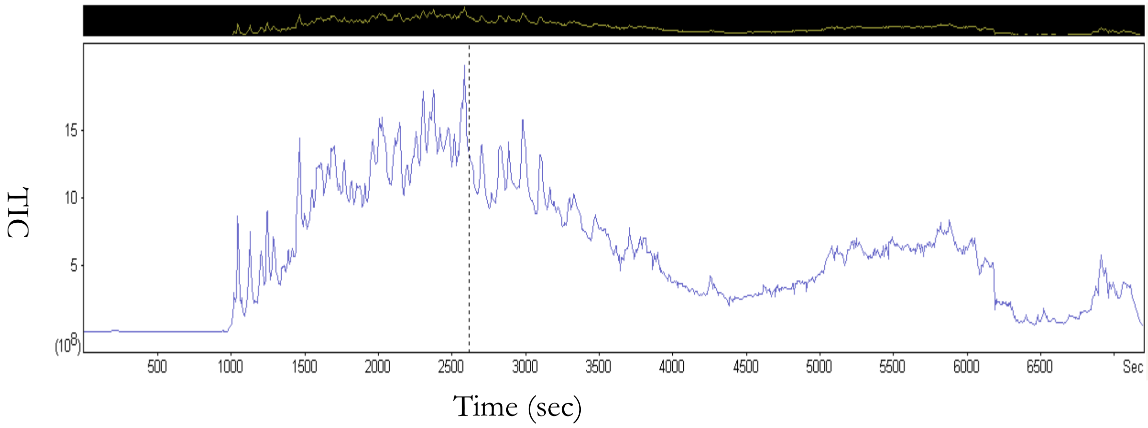

(b)

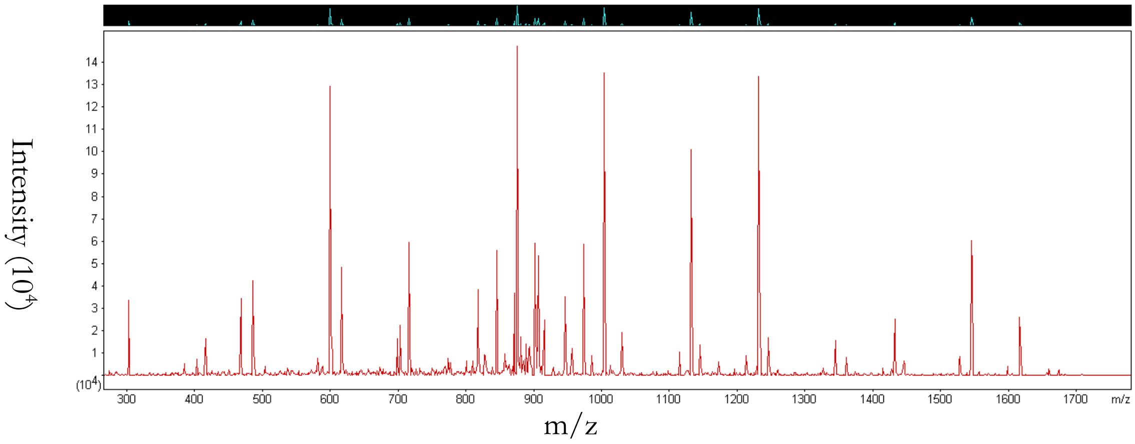

Supplementary Figure 1: LC MS/MS raw data for H37Rv TP injection 1 (a) The elution profile of peptides showing the total ion count across the timeframe with a peak elution between 2,000 and 3,000 seconds; (b) An expanded 2D view of all the peptides eluted at the selected time point of 2,600 sec in the 1D chromatogram.

Drug Response

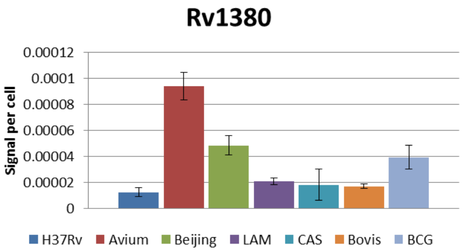

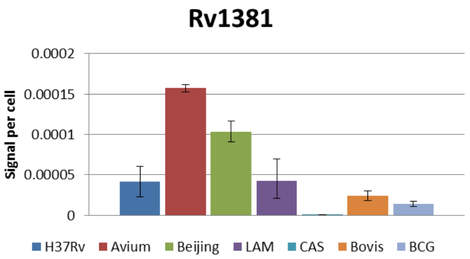

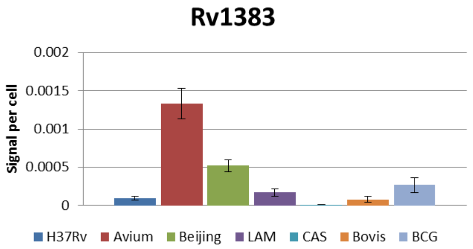

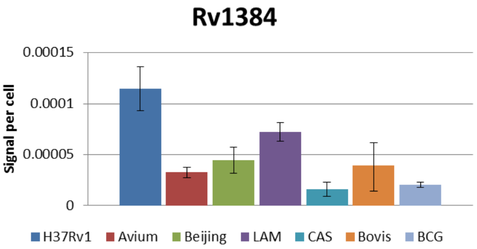

Modulation of immune response

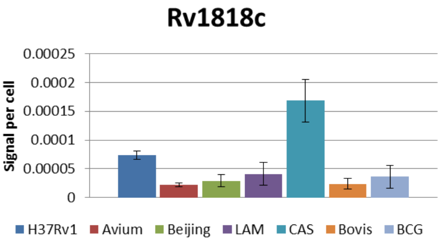

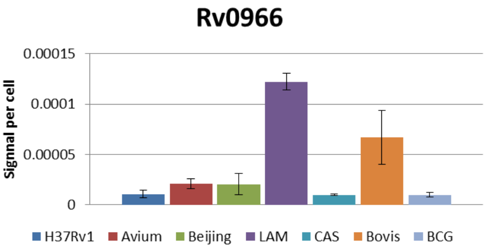

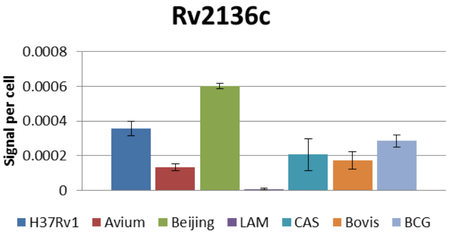

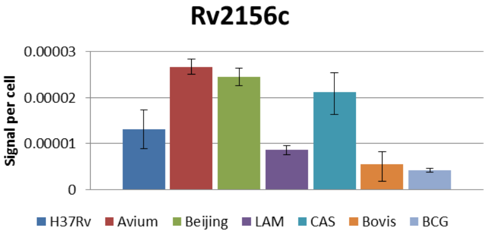

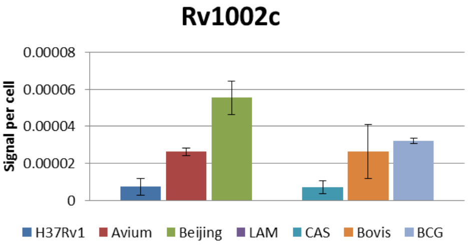

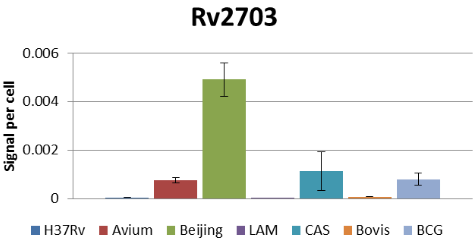

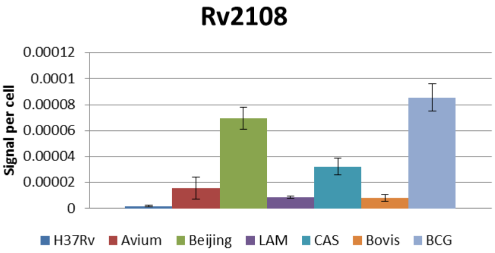

Growth in host

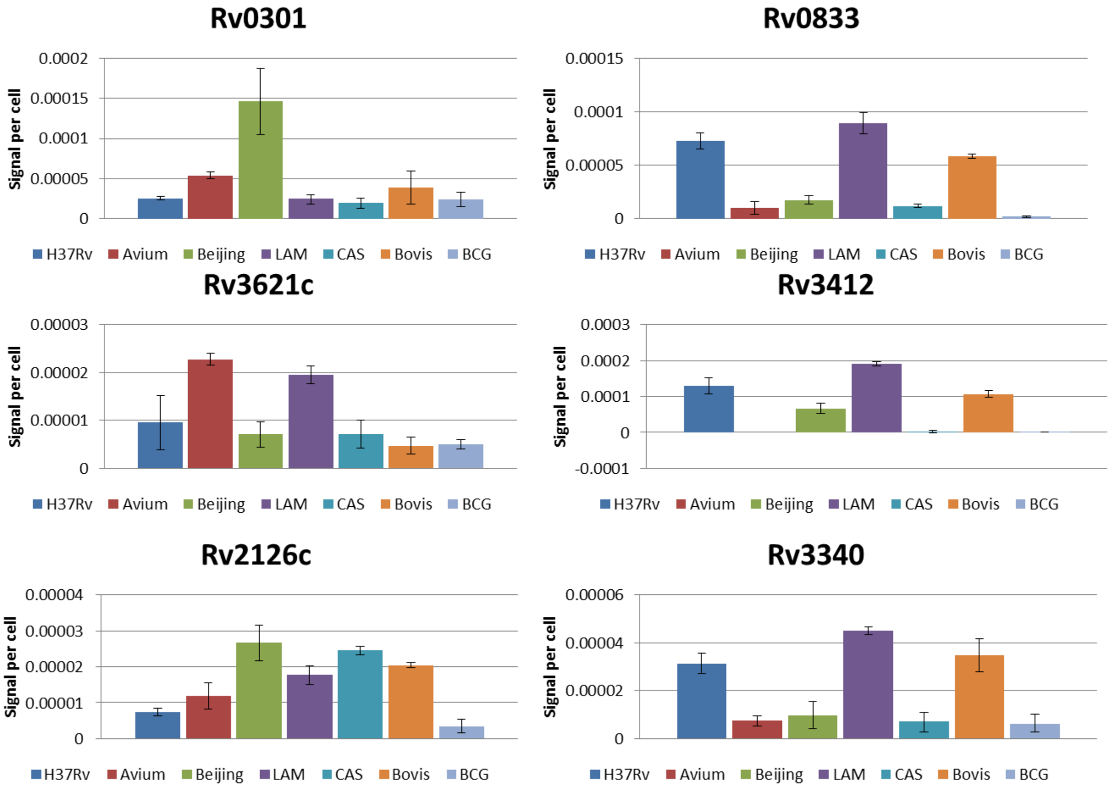

Adaptation to stress

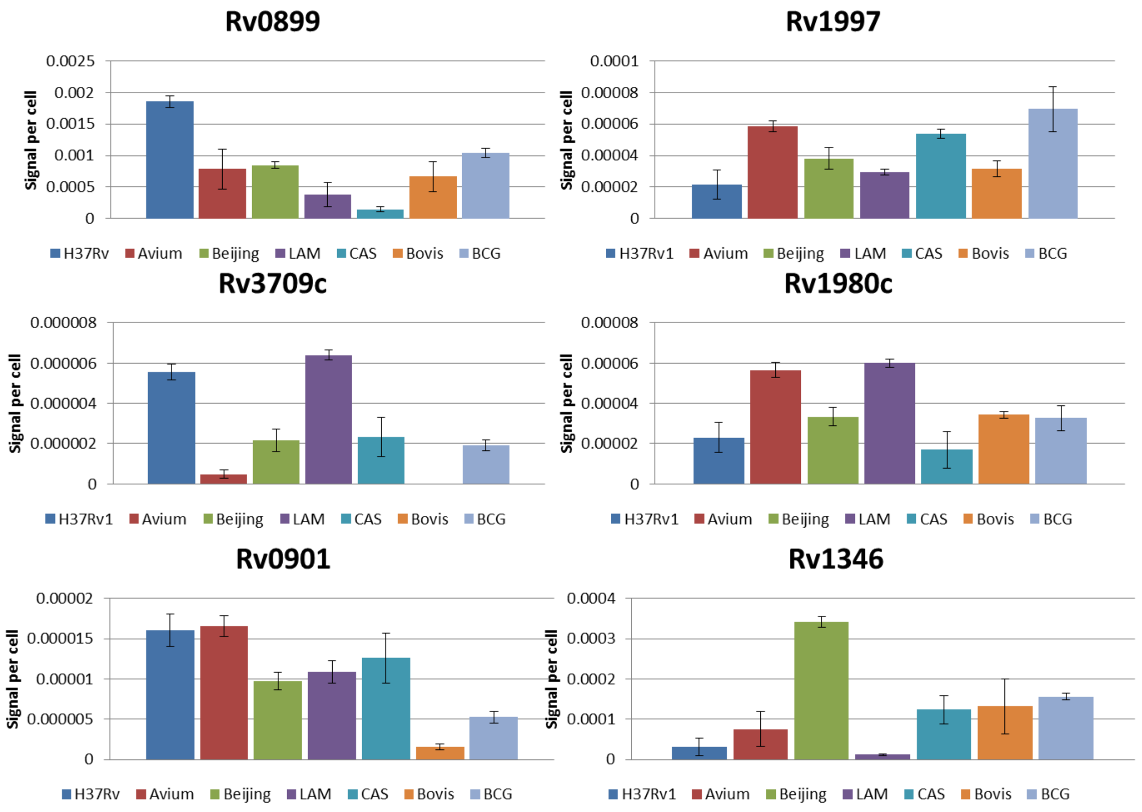

Supplementary Figure 2: Relative quantitation of 23 proteins in in-vitro grown culture. The bar graphs represent signal per cell of each protein per strain as measured by SRM assay using one representative signature peptide
